# Editorial: Risk-stratification in myelodysplastic syndromes

**DOI:** 10.3389/fonc.2022.1095847

**Published:** 2022-12-15

**Authors:** Massimo Breccia, Francesca Vinchi, Massimiliano Bonifacio, Gianni Binotto, Gang Zheng

**Affiliations:** ^1^ Department of Translational and Precision Medicine, Az. Policlinico Umberto I-Sapienza University, Rome, Italy; ^2^ Iron Research Laboratory, New York Blood Center, New York, NY, United States; ^3^ Department Pathology and Laboratory Medicine, Weill Cornell Medicine, New York, NY, United States; ^4^ Department of Medicine, Verona University, Verona, Italy; ^5^ Department of Medicine, Hematology Unit, Padova University Hospital, Padova, Italy; ^6^ Department of Laboratory Medicine and Pathology, Mayo Clinic, Rochester, MN, United States

**Keywords:** myelodysplastic syndromes, prognosis, score systems, NGS, outcome, survival

Prognostic stratification in myelodysplastic syndromes (MDS) has become crucial over the years in defining the best therapeutic strategy to adopt. The incorporation of cytogenetics, percentage of blasts and number and severity of cytopenia allowed, through the IPSS-R, to clinically stratify different categories of patients with different survival. The new IPSS-Molecular (IPSS-M) considers the prognostic role of the somatic mutations found through next generation sequencing (NGS). However, this technique is not feasible everywhere due to the high costs and expertise required. This Research Topic highlights NGS methodology in different MDS subsets as well as possible alternative stratifications. Fang et al applied NGS data to improve prognostic stratification in terms of progression-free survival (PFS) in patients with low-risk MDS. In a cohort of more than 300 patients, incorporation of JAK2, RUNX1 mutations and a blast count greater than 1.5%, in IPSS-R allows better risk stratification and identifying patients who require early therapeutic intervention.

Fattizzo et al. focused on morphological, immunological, and molecular aspects of a retrospective cohort of 226 patients with low-risk MDS. Autoimmunity was detected in 46% of the patients, identifying a subgroup with good response to erythroid stimulating agents (ESAs), whereas 68% of patients tested for somatic mutations had at least one mutation (most frequently ASXL1, TET2, SF3B1, and SRSF2) with, indeed, worse response to ESAs and increased rate of neutropenia. The study suggested that low-risk MDS diversity can be assessed through various clinical and biological features to optimize treatment strategies.

Chen et al. used platelet-large cell ratio (P-LCR) to stratify MDS patients. P-LCR is the percentage of platelets larger than 12 fl, generated by automated counter. A whole cohort of 122 MDS patients was analyzed with statistical differences among P-LCR in low/intermediate-1 compared to intermediate-2/high risk patients. Overall, a low P-LCR was associated to a worse overall survival (OS) but not with progression-free survival (PFS). P-LCR could thus be a valuable immediate prognostic marker for patients with MDS.

In addition, Wang et al. explored the prognostic value of prolonged thrombocytopenia (PT) for MDS patients after hematopoietic stem cells transplant (HSCT). A cohort of 303 MDS patients who underwent HSCT was analyzed: PT is an independent risk factor for worse OS but not for disease relapse. Significant predictive risk factors for PT were grade II-IV acute graft versus host disease (GVHD), extensive chronic GVHD, hemorrhagic cystitis, and cytomegalovirus (CMV) activation. Therefore, risk factors identification for PT is expected to improve the management of MDS patients after allogenic transplant.

Finally, Liang et al developed a novel prognostic score for MDS on a cohort of 201 patients and validated it on a cohort of 115 patients. The new prognostic model has the aim to incorporate somatic mutations among the potential risk factors able to predict OS. Six different parameters were considered as variables to build a nomogram through a “regplot” package: age, bone marrow blast percentage, and 4 different mutations (ETV6, p53, EZH2, ASXL1) retained the strength of prognostic value in multivariate analysis. A prognostic nomogram was then realized and validated with an external cohort. Compared to IPSS and IPSS-R, this nomogram improved the prognostic stratification in the whole analyzed cohort of patients and seems not inferior to the recently developed IPSS-M, proving predictive value in the stratification of MDS patients with high-risk disease and worse survival compared to low risk disease.

The studies presented in this Research Topic highlight the importance of prognostic tools for MDS which include somatic mutations as well as other clinical and laboratory parameters that help dissecting the heterogeneity of this group of diseases, allowing optimizing therapeutic strategies.

## Author contributions

All authors listed have made a substantial, direct, and intellectual contribution to the work and approved it for publication.

